# Neuropeptide-Y causes coronary microvascular constriction and is associated with reduced ejection fraction following ST-elevation myocardial infarction

**DOI:** 10.1093/eurheartj/ehz115

**Published:** 2019-03-11

**Authors:** Neil Herring, Nidi Tapoulal, Manish Kalla, Xi Ye, Lyudmyla Borysova, Regent Lee, Erica Dall’Armellina, Christopher Stanley, Raimondo Ascione, Chieh-Ju Lu, Adrian P Banning, Robin P Choudhury, Stefan Neubauer, Kim Dora, Rajesh K Kharbanda, Keith M Channon, Oxford Acute Myocardial Infarction (OxAMI) Study, Adrian P Banning, Robin P Choudhury, Stefan Neubauer, Kim Dora, Rajesh K Kharbanda, Keith M Channon

**Affiliations:** 1 Department of Physiology, Anatomy and Genetics, Burdon Sandersn Cardiac Science Centre, University of Oxford, Parks Road, Oxford OX13PT, UK; 2 Department of Cardiovascular Medicine, British Heart Foundation Centre of Research Excellence, University of Oxford, John Radcliffe Hospital, Headley Way, Oxford, UK; 3 Department of Pharmacology, University of Oxford, Mansfield Road, Oxford UK; 4 Oxford Acute Vascular Imaging Centre, Department of Cardiovascular Medicine, University of Oxford, John Radcliffe Hospital, Headley Way, Oxford UK; 5 Bristol Heart Institute, Bristol Royal Infirmary, and Faculty of Health Sciences, University of Bristol, Horfield Road, Bristol UK; 6 National Institute for Health Research (NIHR) Biomedical Research Centre, Oxford University Hospitals NHS Foundation Trust, John Radcliffe Hospital, Headley Way Oxford, UK

**Keywords:** Neuropeptide-Y, Myocardial infarction, Percutaneous coronary intervention, Cardiac magnetic resonance imaging, Microvascular function

## Abstract

**Aims:**

The co-transmitter neuropeptide-Y (NPY) is released during high sympathetic drive, including ST-elevation myocardial infarction (STEMI), and can be a potent vasoconstrictor. We hypothesized that myocardial NPY levels correlate with reperfusion and subsequent recovery following primary percutaneous coronary intervention (PPCI), and sought to determine if and how NPY constricts the coronary microvasculature.

**Methods and results:**

Peripheral venous NPY levels were significantly higher in patients with STEMI (*n* = 45) compared to acute coronary syndromes/stable angina ( *n* = 48) or with normal coronary arteries (NC, *n* = 16). Overall coronary sinus (CS) and peripheral venous NPY levels were significantly positively correlated (*r* = 0.79). STEMI patients with the highest CS NPY levels had significantly lower coronary flow reserve, and higher index of microvascular resistance measured with a coronary flow wire. After 2 days they also had significantly higher levels of myocardial oedema and microvascular obstruction on cardiac magnetic resonance imaging, and significantly lower ejection fractions and ventricular dilatation 6 months later. NPY (100–250 nM) caused significant vasoconstriction of rat microvascular coronary arteries via increasing vascular smooth muscle calcium waves, and also significantly increased coronary vascular resistance and infarct size in Langendorff hearts. These effects were blocked by the Y_1_ receptor antagonist BIBO3304 (1 μM). Immunohistochemistry of the human coronary microvasculature demonstrated the presence of vascular smooth muscle Y_1_ receptors.

**Conclusion:**

High CS NPY levels immediately after reperfusion correlate with microvascular dysfunction, greater myocardial injury, and reduced ejection fraction 6 months after STEMI. NPY constricts the coronary microcirculation via the Y_1_ receptor, and antagonists may be a useful PPCI adjunct therapy.

**Figure:**
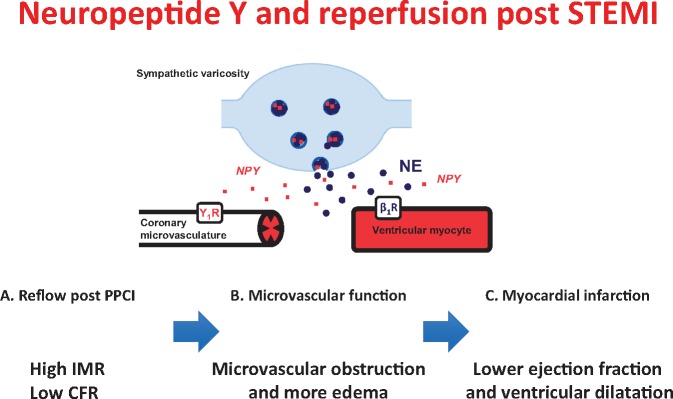

## Introduction

The rapid re-opening and stenting of occluded epicardial coronary arteries via emergency primary percutaneous coronary intervention (PPCI) has revolutionized the treatment of ST-elevation myocardial infarction (STEMI). Despite technical refinements to the procedure and the introduction of numerous antiplatelet and anticoagulant medications, around one-third of patients demonstrate ‘no-reflow’ due to flow limitation in small intramyocardial arteries and arterioles beyond the point of the stenting known as the ‘microcirculation’. This is associated with persistent ST-elevation, larger infarct size, lower ejection fraction, and worse prognosis.^1^ Patients with a high index of microcirculatory resistance (IMR),^2^ or with persistently low coronary flow reserve (CFR)^3^ following PPCI measured using a coronary pressure wire, have larger infarcts and lower ejection fractions. Microvascular obstruction can also be directly imaged using contrast-enhanced cardiac magnetic resonance imaging (CMR) and its presence strongly predicts infarct size and adverse prognosis.^4^ Distal athero-thrombotic embolization from the ruptured plaque and thrombus^5^ may contribute to poor microcirculatory perfusion although clinical trials of thrombectomy at the time of PPCI have failed to demonstrate consistent improvements in outcome.^6^ Functional vasoconstriction in the coronary microvasulature may also contribute to poor microvascular flow, but the mechanisms are poorly understood. Commonly used vasodilator drugs such as adenosine and sodium nitrprusside have not demonstrated clinical benefit,^7^ suggesting that these mechanisms remain to be identified. It is likely that there are reversible as well as irreversible components to microvascular obstruction, the former of which could provide new therapeutic targets.

Acute myocardial infarction is associated with high levels of cardiac sympathetic drive, which is a poor prognostic indicator.^8^ Prolonged sympathetic activation causes the release of the co-transmitter neuropeptide-Y (NPY),^9^ which can cause vasoconstriction in a variety of vascular beds.^10^ When infused directly into coronary arteries in humans, NPY can induce chest pain and ischaemic electocardiogram (ECG) changes presumably from microvascular constriction.^11^ We have shown that peripheral venous levels of NPY are significantly elevated in patients undergoing PPCI following STEMI and remain high for at least 48 h.^12^ Clinical studies before the advent of PPCI and modern medical treatment, have also shown that peripheral venous ‘NPY-like activity’ is elevated during ischaemic events and correlates with 1-year mortality.^13^ However, hepato-mesenteric release also contributes significantly to circulating levels of ‘NPY-like activity’,^14^ making peripheral venous sampling less accurate in determining local cardiac NPY concentrations.

We, therefore, hypothesized that coronary sinus (CS) levels of NPY would provide a close correlation with measurements of reperfusion and microvascular obstruction in STEMI patients undergoing PPCI, and may determine the degree to which myocardial function recovers. Further to investigate possible causation, we tested whether NPY vasoconstricts the coronary microvasculature and increases infarct size in the rat, and then explored the receptor pathways involved to assess whether these may be applicable for human pharmacological intervention.

## Methods

See Supplementary material online for expanded *Methods*. This study complies with the Declaration of Helsinki and was approved by local ethics committee (REC: 10/H0408/24 and 10/H0606/36). All participants gave written informed consent. Patients were recruited as part of the Oxford Acute Myocardial Infarction (OxAMI) study. Animal use complied with the University of Oxford local ethical guidelines and the Animals (Scientific Procedures) Act 1986 (UK).

## Results

### Peripheral venous and coronary sinus neuropeptide-Y levels in patients with normal coronary arteries, stable angina, acute coronary syndromes, and ST-elevation myocardial infarction

45 patients with acute left coronary artery STEMI (presenting throughout the 24-h cycle of clinical activity) underwent peripheral venous and CS blood sampling immediately after completion of PPCI. NPY levels were compared with 48 patients who were pain-free and undergoing non-emergency coronary angiography for stable angina (SA) or acute coronary syndromes (ACS) and a group of 16 patients undergoing elective coronary angiography who were found to have normal coronary arteries (NC). The patients demographics, admission medications, and haemodynamics at the time of angiography are summarized in *Table 1*. Patients were of similar age with similar cardiovascular risk factors, although significantly fewer STEMI patients had a diagnosis of hyperlipidaemia at presentation. Significantly fewer STEMI patients were taking beta-blockers, angiotensin converting enzyme inhibitors/angiotensin receptor antagonists, or a statin at presentation. Moreover, patients presenting with STEMI who were in pain at the time of PPCI had significantly higher heart rate and diastolic blood pressure. As expected, patients with STEMI had the highest peak troponin rise followed by those experiencing SA/ACS with those with NC having minimal troponin rise.

**Table 1:** Patient demographics according to clinical diagnosis (peripheral venous blood)

	NC (*n* = 16)	SA/ACS (*n* = 48)	STEMI (*n* = 45)	*P*-value	*P*-value (NC vs. SA/ACS)	*P*-value (NC vs. S TEMI)	*P*-value (SA/ACS vs. STEMI)
Age (years)	67 ± 12	65 ± 12	63 ± 13	0.39	1.00	0.59	1.00
Males	10/16 (63)	36/48 (75)	35/45 (77.8)	0.48	0.35	0.32	0.81
Cardiovascular risk factors							
Hypertension	13/16 (81)	32/48 (67)	19/45 (42.2)	0.36	0.35	1.00	0.36
Hyperlipidaemia	9/16 (56)	41/48 (85)	16/45 (35.6)	<0.0001	0.031	0.24	<0.0001
Diabetes mellitus	3/16 (19)	10/48 (21)	6/45 (13.3)	0.63	1.00	0.69	0.42
Current smoker	2/16 (13)	13/48 (27)	19/45 (42.2)	0.06	0.32	0.037	0.13
Ex-smoker	8/16 (50)	21/48 (43)	19/45 (42.2)	0.86	0.77	0.77	>0.9999
Family history	8/16 (50)	22/48 (46)	19/45 (42.2)	0.85	0.78	0.77	0.84
Medications on admission							
Beta-blockers	10/16 (63)	27/48 (56)	4/45 (8.9)	<0.0001	0.77	<0.0001	<0.0001
ACE inhibitor/ATR antagonist	11/16 (69)	31/48 (65)	7/45 (15.6)	<0.0001	1.00	0.0002	<0.0001
Statin	14/16 (88)	39/48 (81)	8/45 (17.8)	<0.0001	0.72	<0.0001	<0.0001
Blood pressure and heart rate							
Systolic (mmHg)	130.3 ± 27.8	125.9 ± 22.7	135.3 ± 27.3	0.23	1.00	1.00	0.26
Diastolic (mmHg)	64.1 ± 10.2	69.3 ± 11.1	81.4 ± 17.9	<0.0001	0.78	0.001	0.0005
Heart rate (/min)	66.4 ± 14.4	69.2 ± 13.7	79.6 ± 21.7	0.008	1.00	0.05	0.02
Peak Troponin I (mg/L)	0.3 ± 0.6	3.8 ± 12.4	40.7 ± 16.2	<0.0001	1.00	<0.0001	<0.0001

Values are expressed mean  ±  SD or *n* (%).

ACS, acute coronary syndromes; NC, normal coronary arteries; SA, stable angina; STEMI, ST-elevation myocardial infarction.

**Figure 1:**
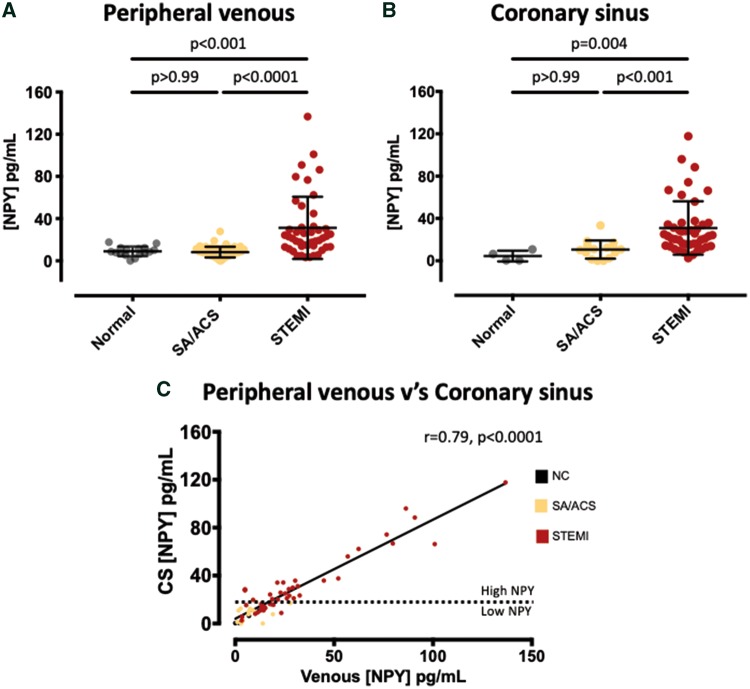
Relationship between peripheral venous and coronary sinus neuropeptide-Y. Peripheral venous (*A*) concentrations of neuropeptide-Y in patients with normal coronary arteries (*n* = 16), stable angina or acute coronary syndromes (*n* = 48), or ST-elevation myocardial infarction (*n* = 45). (*B*) Coronary sinus concentrations of neuropeptide-Y in the same groups (normal coronary arteries, *n* = 4, stable angina or acute coronary syndromes, *n* = 15, ST-elevation myocardial infarction, *n* = 45). (*C*) There is a strong positive correlation between peripheral venous and coronary sinus levels across all patient groups when paired samples were taken (*r* = 0.79, *n* = 64, *P* < 0.0001). Both coronary sinus and peripheral venous levels in the ST-elevation myocardial infarction group were three times higher than those in the stable angina or acute coronary syndromes groups, and this was used to define the lower tertial of coronary sinus neuropeptide-Y levels in the ST-elevation myocardial infarction group as being low, and the middle and upper tertial as being high as illustrated by the dotted line in (*C*). NSTEMI, non-ST-elevation myocardial infarction; UA, unstable angina.

The levels of peripheral venous and CS NPY in the three groups are summarized in *Figure 1*. Overall paired CS and peripheral venous NPY were significantly positively correlated (*r* = 0.79, *n* = 64, *P* < 0.0001) as shown in *Figure 1C*. Peripheral venous and CS levels of NPY were similar in the SA/ACS and NC groups, as shown in *Figure 1A and B*. Patients with STEMI had three-fold higher CS NPY levels than those with SA/ACS or NC. We used this to define the lower tertile of the STEMI group as having low CS NPY levels (*n* = 15), similar to those with NC and SA/ACS [12.9 (9.0–14.9) vs. NC/SA/ACS: 10.0 (1.8–12.4 pg/mL)], and the middle and upper tertiles as having high CS NPY levels (*n* = 30), shown by the dotted line in *Figure 1C*.

### Invasive measures of microvascular function in STEMI patients with high vs. low coronary sinus neuropeptide-Y levels

The baseline characteristics of STEMI patients with low and high CS NPY levels are summarized in *Table 2*. The two groups were well matched in terms of age, sex, cardiovascular risk factors, medications, and haemodynamics on admission. They had similar TIMI flow and pain to balloon time at presentation. There was no significant correlation between CS NPY levels and pain to balloon time (*r* = 0.24, *P* = 0.1). Although levels of CS NPY were significantly different between the groups, levels of CS endothelin-1 were similar and there was no significant correlation between CS NPY and endothelin-1 levels (*r* = 0.001, *P* = 0.81).

**Table 2:** Clinical characteristics according to coronary sinus neuropeptide-Y level

	Low CS NPY	High CS NPY	*P*-value
	(*n* = 15)	(*n* = 30)	
Age (years)	58.4 ± 12.9	64.5 ± 12.9	0.14
Males	13/15 (86.7)	22/30 (73.3)	0.46
Cardiovascular risk factors			
Hypertension	5/15 (33.3)	14/30 (46.7)	0.53
Hyperlipidaemia	7/15 (46.7)	9/30 (30)	0.33
Diabetes mellitus	3/15 (20)	3/30 (10)	0.65
Current smoker	7/15 (46.7)	12/30 (40)	0.75
Ex-smoker	7/15 (46.7)	12/30 (40)	0.75
Family history	6/15 (40)	13/30 (43.3)	1.00
Medications on admission			
Beta-blockers	0/15 (0)	4/30 (13.3)	0.28
ACE inhibitor/ATR antagonist	2/15 (13.3)	5/30 (16.7)	1.00
Statin	3/15 (20)	5/30 (16.7)	1.00
BP and heart rate at presentation			
Systolic BP (mmHg)	135.1 ± 4.1	135.4 ± 5.8	0.97
Diastolic BP (mmHg)	82.7 ± 4.0	80.7 ± 3.5	0.71
Heart rate (/min)	78.4 ± 4.0	80.1 ± 4.5	0.77
Pain to balloon time (min)	368 ± 85	244 ± 47	0.21
Infarct artery			
LAD	11/15 (73.3)	22/30 (73.3)	1.00
LCx/Int	4/25 (26.7)	8/30 (26.7)	1.00
TIMI flow at presentation			
0	11/15 (73.3)	24/30 (80)	0.71
I	1/15 (6.7)	2/30 (6.7)	1.00
II	2/15 (13.3)	3/30 (10)	1.00
III	1/15 (6.7)	1/30 (3.3)	1.00
Peak Troponin I (mg/L)	40.8 ± 16.7	40.6 ± 16.2	0.97
Coronary sinus NPY concentration (pg/mL)	**12.9 (9.0–14.9)**	**29.3 (23.6–51.4)**	**<0.00001**
Coronary sinus endothelin-1 concentration (fmol/mL)	3.3 (2.1–22.3)	5.0 (2.0–10.7)	0.87

Values are mean ± SD, median (interquartile range), or *n* (%). Statistically significant results are in bold.

LAD, left anterior descending artery; LCx, left circumflex artery; Int, intermediate artery.

Immediately after stent implantation and post-dilatation, invasive measurements of coronary microcirculatory function were taken using a pressure wire. Coronary haemodynamic measures are summarized in *Table 3*. Patients with high CS NPY levels had significantly lower CFR and a significantly higher IMR. This relationship did not reach significance when considering low vs. high peripheral venous NPY (CFR 1.84 ± 0.88 vs. 1.52 ± 0.55, *P* = 0.22; IMR 35.2 ± 18.7 vs. 43.2 ± 31.2, *P* = 0.29) or low vs. high CS endothelin-1 (CFR 1.62 ± 0.75 vs. 1.63 ± 0.66, *P* = 0.94; IMR 50.1 ± 38.3 vs. 35.7 ± 19.5, *P* = 0.19). In a subset of patients a collateral pressure index was also measured, although there was no significant difference between patients with low v’s high CS NPY (0.16 ± 0.09, *n* = 7 vs. 0.15±0.07, *n* = 10).

**Table 3:** Coronary physiology according to coronary sinus neuropeptide-Y levels

Coronary haemodynamics	Low CS NPY	High CS NPY	*P*-value
	(*n* = 15)	(*n* = 30)	
Baseline fractional flow reserve	0.91 ± 0.06	0.92 ± 0.05	0.37
Hyperaemic fractional flow reserve	0.87 ± 0.08	0.90 ± 0.07	0.11
Baseline transit time (s)	0.87 ± 0.47	0.77 ± 0.37	0.45
Hyperaemic transit time (s)	0.48 ± 0.24	0.60 ± 0.40	0.24
Baseline distal pressure (mmHg)	84.5 ± 14.5	90.9 ± 16.4	0.19
Hyperaemic distal pressure (mmHg)	**68.0** ± **13.3**	**77.0** ± **14.0**	**0.04**
Coronary flow reserve	**1.94** ± **0.81**	**1.47** ± **0.55**	**0.03**
Index of microcirculatory resistance	**30.9** ± **12.2**	**45.3** ± **31.9**	**0.03**

Values are expressed as mean  ±  SD. Statistically significant results are in bold.

### Imaging measures of microvascular function and cardiac recovery in STEMI patients with high vs. low coronary sinus neuropeptide-Y levels

Two days following PPCI, all patients underwent CMR and there was no difference in the time to scan between those patients with low vs. high CS NPY [41.5 (33.8–47.3) vs. 48.0 (28.5–49.0) h, *P* = 0.38]. This demonstrated similar left ventricular dimensions and ejection fraction in the high and low CS NPY groups at this stage. However, there was a significantly larger volume of myocardial oedema in those patients with high CS NPY levels and a significantly greater volume of microvascular obstruction. Six months following STEMI a further CMR scan demonstrated a significantly lower ejection fraction in those with high CS NPY compared to those with low CS NPY levels (50.5 ± 11.8, *n* = 12, vs. 61.7 ± 3.8%, *n* = 23, *P* < 0.0001) and significantly dilated left ventricular volumes as shown in *Table 4*. Overall CS NPY levels were positively correlated with infarct size (by late gadolinium enhancement) at 6 months (*r* = 0.46, *P* = 0.01) as was acute microvascular obstruction (*r* = 0.55, *P* = 0.003). However, on multivariate analysis, acute microvascular obstruction was an independent predictor of infarct size (*P* = 0.01) above CS NPY, in keeping with CS NPY causing larger infarcts via worsening microvascular obstruction. The relationship with infarct size, ejection fraction and left ventricular volumes did not reach significance when considering high vs. low peripheral venous NPY (ejection fraction 51.7 ± 11.6 vs. 58.8 ± 9.6%, *P* = 0.12).

**Table 4:** Cardiac magnetic resonance imaging post-ST-elevation myocardial infarction according to coronary sinus neuropeptide-Y levels

Cardiac MRI	Low CS NPY	High CS NPY	*P*-value
	(*n* = 12)	(*n* = 23)	
Post-PPCI			
Ejection fraction (%)	47.3 ± 8.5	44.6 ± 10.0	0.40
End-diastolic volume (mL)	142.9 ± 48.5	162.9 ± 45.4	0.25
End-systolic volume (mL)	78.1 ± 38.2	92.4 ± 35.8	0.30
Late Gd enhancement (%)	25.2 ± 13.6	34.1 ± 16.3	0.10
Haemorrhage (%)	1.0 ± 1.9	3.6 ± 6.1	0.07
Ventricular oedema (%)	**33.4** ± **14.0**	**44.5** ± **16.2**	**0.048**
Microvascular obstruction (%)	**0.22** ± **0.12**	**1.62** ± **2.52**	**0.02**
6 months			
Ejection fraction (%)	**61.7** ± **3.8**	**50.5** ± **11.8**	**<0.001**
End-diastolic volume (mL)	**140.9** ± **28.0**	**173.0** ± **47.3**	**0.038**
End-systolic volume (mL)	**54.6** ± **14.7**	**87.6** ± **38.5**	**0.003**

Values are expressed as mean  ±  SD. Statistically significant results are in bold.

Gd, gadolinium; MRI, magnetic resonance imaging.

### The action of neuropeptide-Y on microvascular coronary arteries and Langendorff perfused hearts

**Figure 2:**
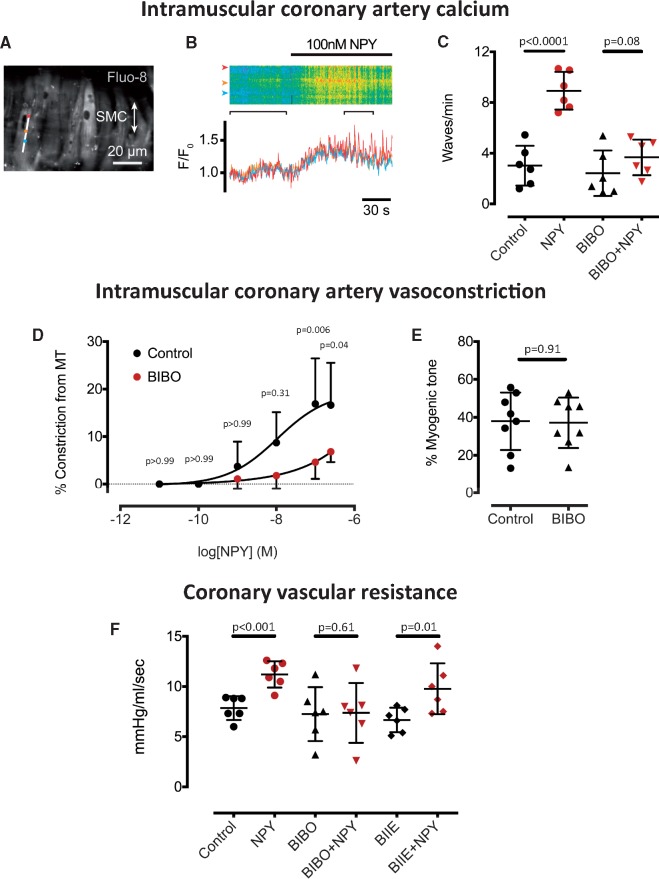
Microvascular coronary artery vasoconstriction with neuropeptide-Y. Neuropeptide-Y (100 nM, *n* = 6) increases calcium wave frequency in vascular smooth muscle cells of microvascular coronary arteries measured using Fluo-8. (*A*) The location of a smooth muscle cell line scan (*B*) A raw data trace of normalized Fluo-8 florescence (F/F_0_) in response to neuropeptide-Y over the line scan and at three separate points (red, orange, and blue). This is prevented by the Y_1_ receptor antagonist (BIBO3304, 1 μM, *n* = 6) as shown in (*C*). (*D*) Dose-response curve to neuropeptide-Y (*n* = 6) demonstrating vasoconstriction in isolated, pressurized microvascular coronary arteries. This is prevented by the Y_1_ receptor antagonist (BIBO3304, 1 μM, *n* = 6), without altering basal myogenic tone (*E*). (*F*) Neuropeptide-Y (250 nM, *n* = 6) causes a significant increase in coronary vascular resistance in the Langendorff heart and this is prevented by the Y_1_ receptor antagonist BIBO3304 (1 μM, *n* = 6), but not the Y_2_ receptor antagonist BIIE0246 (1 μM, *n* = 6).

**Figure 3:**
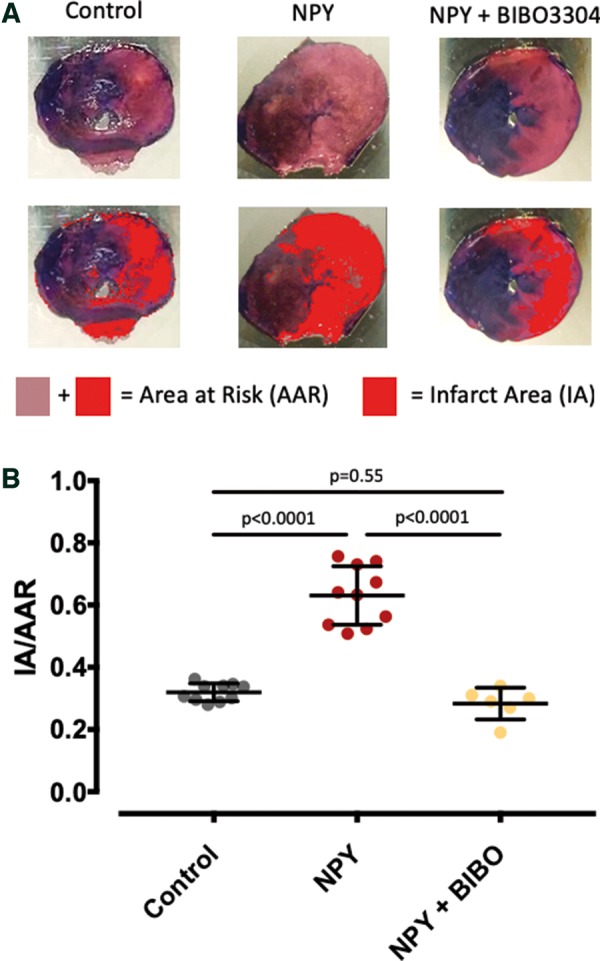
Neuropeptide-Y Y_1_ receptor antagonism and infarct size. (*A*) Examples of single ventricular slices at the mid ventricular level which have undergone staining with Evans blue (to identify tissue outside the area at risk) and triphenyltetrazolium chloride to identify the infarct area. The later is very pale pink in the top images and red in the bottom images as identified using ImageJ software. (*B*) Neuropeptide-Y (250 nM, *n* = 10) significantly increases infarct area in relation to the area at risk during ischaemia reperfusion in the Langendorff heart compared to control (*n* = 10). This is blunted by the Y_1_ receptor antagonist BIBO3304 (1 μM, *n* = 6). The area at risk in relation to the total heart area was similar between groups (control 60.8 ± 7.4%, *n* = 10; neuropeptide-Y 60.7 ± 9.2%, *n* = 10; BIBO3304 + NPY 62.3 ± 10.7% *n* = 6).

In order to investigate the mechanistic importance of high myocardial NPY in mediating coronary microvascular dysfunction, we tested the direct effects of NPY on microvascular coronary arteries. NPY (100 nM, *n* = 6) caused calcium waves in vascular smooth muscle cells (*Figure 2A*–*C*) and dose dependent vasoconstriction of isolated microvascular coronary arteries (significant at 100 and 250 nM, *n* = 6, *Figure 2D*), which could be prevented by the Y_1_ receptor antagonist BIBO3304 (1 μM) without changing myogenic tone (*Figure 2E*). In the Langendorff perfused heart, NPY (250 nM, *n* = 6) caused a significant increase in coronary vascular resistance (*Figure 2F*) that could also be prevented by the Y_1_ receptor antagonist BIBO3304 (1 μM, *n* = 6) but not the Y_2_ receptor antagonist BIIE0246 (1 μM, *n* = 6). NPY (250 nM, *n* = 10) also significantly increased infarct size in relation to the area at risk during left coronary artery ischaemia reperfusion compared with control hearts (*n* = 10), and this could also be prevented by the Y_1_ receptor antagonist BIBO3304 (1 μM, *n* = 6) as shown in *Figure 3*.

### Neuropeptide-Y Y_1_ receptor is present in the human coronary microvasculature

**Figure 4:**
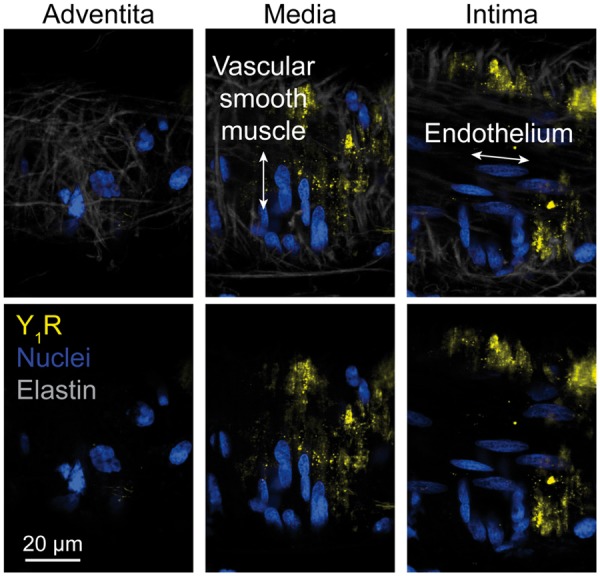
Human coronary microvasculature Y_1_ receptor expression. Neuropeptide-Y Y_1_ receptor expression (in yellow) on vascular smooth muscle cells within the media of a pressurized human coronary micro-artery. Lack of staining in the adventitia and intima of the same vessel is shown for comparison. Nuclear staining in blue, elastin in grey. Representative of three arteries.

We obtained samples of human myocardium during cardiothoracic surgery from which we dissected coronary micro-arteries. Immunohistochemistry demonstrated the presence of the Y_1_ receptor on vascular smooth muscle cells in the media of the vessels as shown in *Figure 4*.

**Take home figure:**
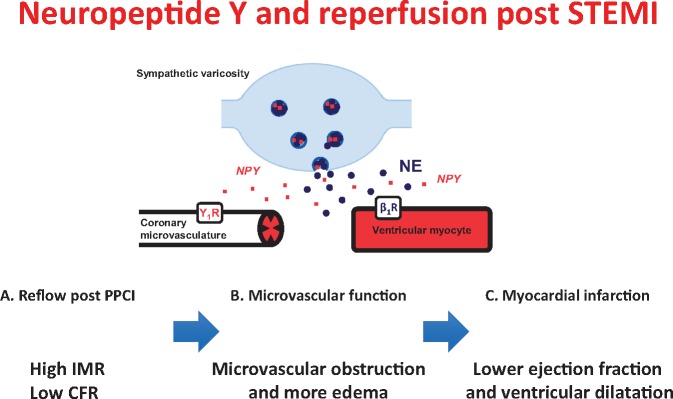
Neuropeptide-Y (NPY) following primary percutaneous coronary intervention (PPCI) for ST-elevation myocardial infarction (STEMI) causes vasoconstriction of the coronary microvasculature and is associated with a high index of microcirculatory resistance (IMR) and low coronary flow reserve (CFR), leading to microvascular obstruction, edema and eventually a lower ejection fraction and ventricular dilatation.

## Discussion

This is the first study to measure CS NPY levels in patients undergoing PPCI and demonstrate a link with microvascular obstruction, infarct size, and subsequent cardiac recovery at 6 months in terms of ejection fraction. In addition it is the first study to show that NPY constricts the coronary microvasculature via Y_1_ receptor dependent calcium mobilization and demonstrate the utility of blocking this receptor in reducing coronary vascular resistance and limiting infarct size. Importantly, the Y_1_ receptor is expressed on vascular smooth muscle cells in the media of human coronary micro-arteries making it a suitable target for pharmacological intervention.

### Coronary sinus and peripheral venous neuropeptide-Y levels in patients undergoing coronary angiography

The main source of circulating NPY is sympathetic nerve terminals and the adrenal medulla and NPY can act as a local neuromodulator of several aspects of cardiac function.^10^ NPY may be involved in the pathogenesis of atherosclerosis,^15^ in addition to maintaining cardiac contraction, promoting ventricular hypertrophy,^10^ and reducing parasympathetic nerve activity.^16^ It may also be taken up into megakaryocytes and released at sites of vascular remodelling as well as in the endothelium itself which also contains dipeptidyl peptidase. There may, therefore, be local autocrine NPY systems at sites of angiogenesis and vascular remodelling.^17^ Animal studies suggest that cardiac NPY is released from sympathetic nerves during experimentally induced myocardial infarction.^18^ Early studies in the late 1980s, before modern interventional and pharmacological treatment of STEMI have shown that peripheral venous ‘NPY-like activity’ is elevated during ischaemic events and correlates with 1-year mortality.^13^ However, hepato-mesenteric release also contributes significantly to circulating levels of ‘NPY-like activity’,^14^ making peripheral venous sampling less accurate in reflecting cardiac NPY levels. These early studies only measured ‘NPY like activity’ with very high limits of detection (>90 pg/mL compared to our 2–3 pg/mL). Our assay has 0% cross-reactivity with structurally similar peptides such as peptide YY, pancreatic polypeptide, gastric inhibitory polypeptide, ghrelin, proinsulin, or glucagon. In patients with normal coronary arteries, we measure CS NPY levels of 4.5 ± 2.5 pg/mL. By comparison, the median peripheral venous NPY level in 303 normal adult subjects (using an assay with a similar level of detection to ours and minimal cross-reactivity) is <2 pg/mL.^19^

STEMI patients had higher NPY levels and were also more hypertensive and tachycardic than patients with SA/ACS and NC who were pain-free at the time of their non-emergency procedure. This is unsurprising given that STEMI patients were suffering from chest pain and would have high levels of sympathetic drive. NPY released during these conditions can also inhibit parasympathetic neurotransmission and the ability of the vagus to reduce heart rate.^9^^,^^16^ Elevated NPY levels have also been observed in animal models^20^ and patients^21^ with essential hypertension. For comparison, venous NPY levels in patients presenting with Takotsubo cardiomyopathy with severely impaired systolic function and massive catecholamine release were around 186 pg/mL.^22^

In the anesthetized dog, direct stimulation of the cardiac sympathetic innervation leads to the appearance of NPY in CS blood.^23^ In patients with cardiac failure or patients with normal hearts undertaking exercise, cardiac release contributes significantly more to circulating peripheral venous levels than at rest.^14^ Whilst peripheral venous and CS levels of NPY are correlated across all three patient groups in our study, this is driven by the very high levels of NPY measured in the STEMI group where cardiac NPY release is likely to have equilibrated with peripheral circulating levels by the time of blood sampling. It is interesting to note that peripheral venous NPY levels are not significantly different in patients with NC and ACS/SA, whereas CS NPY levels are higher in the ACS/SA group compared to patients with NC. Moreover, whilst high CS levels of NPY in the STEMI groups correlated with indices of reperfusion and 6 month ejection fraction, this relationship did not reach significance for peripheral venous levels, suggesting that CS levels may give a more accurate reflection of the NPY concentration to which the coronary microvasculature is exposed and subsequently its behaviour.

### Coronary sinus neuropeptide-Y and measures of reperfusion and cardiac recovery

Contrast-enhanced CMR is considered the gold-standard imaging to assess microvascular obstruction following myocardial infarction, and strongly predicts infarct size and prognosis.^4^ Studies have also correlated an IMR >40 with CMR measures of oedema and microvascular obstruction as well as a worse ejection fraction and ventricular dilatation at 6 months.^24^ CMR measures of microvascular obstruction are also associated with lower CFR.^25^ Patients with high CS NPY levels have significantly higher IMR, lower CFR, and evidence of microvascular obstruction and worse ejection fraction at 6 months on CMR compared to patients with low CS NPY levels. However, the same relationship did not reach significance for high vs. low peripheral venous NPY levels, although there was a strong trend. In a separate cohort of STEMI patients, we have found that those with angiographic no-reflow, lack of electrocardiographic ST-resolution, CFR <1.5, or IMR >33 had significantly higher peripheral venous NPY levels over the first 48 h from admission^12^ although we did not measure CS levels or assess subsequent myocardial damage and prognosis.

### Mechanism of microvascular vasoconstriction

We hypothesize that maintained microvascular vasoconstriction post-PPCI may contribute to a larger infarct size, worse left ventricular function, and poorer prognosis and that NPY may be a key mediator of this. Other substances have also been implicated including endothelin-1,^26^ thromboxane-A2, and B-type natriuretic peptide.^1^ The effect of NPY on small to medium arteries has been studied in a variety of vascular beds^10^ although little is known about the action of NPY in the coronary circulation. NPY produces vasoconstriction in human epicardial coronary arteries by potentiating norepinephrine mediated vasoconstriction.^27^ However, whether NPY vasoconstricts the coronary microvasculature (which lack alpha_1_ adrenergic receptors) has not previously been studied, and these vessels are key to reperfusion post-PPCI. We directly demonstrate that NPY causes a dose-dependent vasoconstriction of microvascular coronary arteries and a rise in coronary vascular resistance in the whole heart.

There are several potential mechanisms by which NPY may induce vasoconstriction including:

- Inhibiting cAMP dependent vasodilatory signalling (e.g. beta_2_ adrenergic receptors) via Y_1_ receptor coupling to inhibitory G proteins.^28^
- Direct mobilization of sarcoplasmic reticulum calcium through a phospholipase C—IP_3_ dependent pathway coupled to the Y_1_ receptor.^28^
- Indirectly through the release of endothelin-1 via the Y_2_ receptor.^29^

Endothelin-1 is a potent vasoconstrictor of small resistance vessels in the coronary circulation and several studies have found an association between peripheral venous endothelin-1 levels and coronary no-reflow post-STEMI.^26^ Although NPY has been shown to cause endothelin-1 release in endocardial endothelial cells via a Y_2_ receptor dependent pathway,^29^ we observe no correlation between CS NPY and endothelin-1 levels. Moreover, the increase in coronary vascular resistance in response to NPY could not be prevented by a Y_2_ receptor antagonist. A Y_1_ receptor antagonist did not on its own cause vasodilatation or decrease coronary vascular resistance suggesting no background beta_2_ receptor stimulation making G_i_ signalling unlikely. In microvascular arteries, we directly observe the mobilization of intracellcular calcium stores suggesting that Y_1_ receptor signalling is most likely coupled to a phospholipase C-IP_3_ dependent pathway.

### Limitations

This is a small mechanistic study that lacks statistical power and studies in large cohorts will be required to further investigate the relationship with overall prognosis. Given the positive correlation between peripheral venous and CS NPY levels, it may be that the same associations we describe for CS NPY also apply to peripheral venous levels if there was a larger sample size. We also did not measure cardiac NPY release via a CS-arterial NPY difference. By the time of sample acquisition, CS and venous NPY levels appear to have equilibrated and therefore a CS-arterial difference will have a low sensitivity. The coronary microvasculature post-PPCI will be exposed to NPY released locally as well as from circulating blood, and the combined concentration will determine its overall physiological response. We feel that this is best reflected in the overall CS concentration rather than a CS-arterial difference.

### Clinical implications

Given our clinical observations regarding CS NPY levels in STEMI patients, the fact that the Y_1_ receptor antagonist BIBO3304 could limit infarct size during ischaemia reperfusion in the rat, and the presence of the Y_1_ receptor on human coronary micro-arteries, we speculate that Y_1_ receptor antagonism may be beneficial post-revascularization by PPCI. This may help relieve microvascular constriction, restore blood flow, minimize infarct size, and improve ejection fraction if there is a reversible component to the obstruction. It is also possible that NPY may play a role in the remodelling process itself.^10^ The competitive Y_1_ receptor antagonist AR-H040922 has been administered as an intravenous infusion in patients with SA but did not influence exercise induced ischaemia at the dose used.^30^ This is unsurprising given that SA is due to flow limiting epicardial coronary artery stenosis, rather than microvascular dysfunction. Whether this or similar compounds can improve microvascular function, reduce infarct size, and improve prognosis in the context of PPCI remains to be established.

## Supplementary Material

ehz115_Supplementary_Data
