# Supplementary material for: Neuropeptide-Y causes coronary microvascular constriction and is associated with reduced ejection fraction following ST-elevation myocardial infarction
Source: Eur Heart J. 2019 Mar 11;40(24):1920–9. doi: 10.1093/eurheartj/ehz115 (PMC6588241; doi:10.1093/eurheartj/ehz115)
Supplement: ehz115_Supplementary_Data [file ehz115_supplementary_data.docx]

**Neuropeptide-Y causes coronary microvascular constriction and is associated with reduced ejection fraction following ST-elevation myocardial infarction.**

**Brief title:** Neuropeptide-Y and microvascular perfusion in STEMI

Neil Herring^1,2*^, Nidi Tapoulal^1^, Manish Kalla^1,2^, Xi Ye^3^, Lyudmyla Borysova^3^, Regent Lee^2^, Erica Dall’Armellina^2,4^, Christopher Stanley^3^, Raimondo Ascione^5^, Chieh-Ju Lu^1^, ‘Oxford Acute Myocardial Infarction (OxAMI) Study’, Adrian P. Banning^2,6^, Robin P. Choudhury^2,4^, Stefan Neubauer^2,6^, Kim Dora^3^, Rajesh K. Kharbanda^2,6^, & Keith M. Channon^2,6^.

**Online methods supplement**

*Study population*

Patients with STEMI[^1^](#_ENREF_1) who underwent PPCI at Oxford University Hospitals NHS Foundation Trust were prospectively enrolled. Exclusion criteria were symptom duration >12 hours, the presence of cardiogenic shock or contraindication to MRI. Another group of patients undergoing non-emergency angiography for stable angina (SA) and acute coronary sydromes (ACS) who were pain free at the time of intervention were recruited and grouped for analysis as they had similar NPY levels, along with a group undergoing non-emergency coronary angiography who were found to have normal coronary arteries (NC). The study complies with the Declaration of Helsinki and was approved by local ethics committee (REC: 10/H0408/24). Verbal assent at the time of emergency PCI was followed by informed written consent, before data and samples were analysed. PPCI was performed according to contemporary international guidelines[^2^](#_ENREF_2). Patients were recuited as part of the Oxford Acute Myocardial Infarction (OxAMI) study between 2010 and 2014.

*Invasive assessment of the coronary microcirculation*

A pressure wire (Certus, Abbott) was placed in the distal third of the infarct-artery to perform invasive assessment of the coronary microcirculation immediately after stent implantation and post-dilatation in STEMI patients. CFR and IMR were measured as previously described[^3^](#_ENREF_3). Briefly, the mean transit time (Tmn) was calculated from three injections of 5 mL room-temperature saline through the guiding catheter. Tmn was recorded at baseline and after induction of hyperemia with intravenous adenosine infusion (140 µg/kg/min) into the right femoral vein. Simultaneous measurements of mean aortic pressure (Pa, by guiding catheter) and mean distal coronary pressure (Pd, by pressure wire) were made in the resting and maximal hyperemic states. CFR was calculated as the ratio of the transit times at baseline and hyperemia. IMR is defined as the simultaneously measured distal coronary pressure multiplied by the hyperemic Tmn (mmHg.seconds). Fractional flow reserve (FFR) is defined as the ratio of the pressure distal to a lesion relative to the pressure proximal to it during maximal hyperemia. In a subset of patients (n=17), a coronary wedge pressure (Pcw) was measured immediately after post-dilation of the stent along with central venous pressure (Pcv) via the venous blood sampling catheter. A collateral pressure index was calculated as (Pcw – Pcv)/(Pa – Pcv) as described previously[^4^](#_ENREF_4).

*Blood sampling*

The coronary sinus was cannulated using a 6F catheter via the right femoral or antecubital vein immediately following PPCI and venous blood samples taken from the CS and peripheral vein sequentially, typically within a few seconds of each other, as described previously[^5^](#_ENREF_5). Patients with left coronary infarcts only were included as venous drainage from the right coronary artery is also via the thebesian system[^6^](#_ENREF_6). Blood was collected using standardized tubes containing dipotassium ethylenedinitro tetraacetic acid, centrifuged at 9000 g for 1 minute before being immediately frozen in liquid nitrogen and stored at −80 °C. NPY and endothelin-1 were measured using commercially available ELISAs (EZHNPY-25K, QET00B) according to manufacturer's instructions. Cardiac Troponin I measurements were performed using automated chemiluminescent immunoassay techniques on the Siemens ADVIA Centaur.

*Cardiac magnetic resonance imaging*

Patients underwent CMR 2 days and 6 months following PPCI using a 3 Tesla scanner (either MAGNETOM TIMTrio or MAGNETOM Verio, Siemens). Left ventricular function and myocardial injury were assessed using Steady State Free Precession, T2-prepared and late gadolinium enhancement imaging as described previously[^7^](#_ENREF_7).

*Isolated microvascular coronary artery myography*

Animal use complied with the University of Oxford local ethical guidelines and the Animals (Scientific Procedures) Act 1986 (UK). Intramyocardial (septal) coronary arteries with smooth muscle layers 2 cells thick and external diameter <250 μm were dissected form adult male Sprague-Dawley rats and a small segment (2 mm) removed and mounted in a pressure myograph. The solution temperature was raised to 37 °C, and the artery pressurized to 80 mmHg.

*Isolated microvascular coronary artery calcium imaging*

Once mounted on the myograph, intraluminal pressure was lowered to ~10 mmHg and the lumen was perfused for ~20 minutes with MOPS-buffered solution containing 0.0625% Pluronic F-127 and 12 μM fluo-8, AM. After the dye was washed out, the artery was re-pressurized to 80 mmHg and left for 15-minutes to allow de-esterification. The artery was excited at ~488 nm and emitted light collected at ~505 nm with an Olympus confocal microscope using a 40x/1.15 water-immersion objective. Recordings of vascular smooth muscle calcium events were acquired at ~3 Hz.

*Coronary vascular resistance in the Langendorff perfused heart*

Following thoracotomy the heart was removed and placed in ice-cold heparinised Tyrode’s solution (50 units/ml) before mounting on a cannula to establish retrograde perfusion via the ascending aorta with constant flow (10 ml/minute). The Tyrode’s solution was filtered before passing through two oxygenators and a bubble trap and maintained at 36-37 °C. An in-line pressure transducer was used to record perfusion pressure. Experimental protocols commenced after 30 minutes equilibration. Coronary vascular resistance was calculated by dividing perfusion pressure (mmHg) by coronary flow rate (ml/minute).

*Infarct size following ischaemia reperfusion in the Langendorff perfused heart*

Langendorff perfusion was established under constant pressure (60 mmHg) at 36-37 °C. Experimental protocols commenced after 30 minutes equilibration. 30 minutes of ischaemia was induced via a suture around the proximal left coronary artery tied against a polyethylene tube to allow coronary blood flow to be re-established. After 60 minutes of reperfusion, the suture was retied and 2 ml of 0.25% w/v Evans blue in phosphate buffered saline was perfused via the aortic canula. The ventricle was then removed, placed in a tissue slicing matrix, covered in parafilm and stored at -80 °C for 10 minutes . The heart was then sliced into 2 mm sections and the ventricle stained with 10 ml of 1% w/v triphenyltetrazolium chloride (TTC) at 37 °C for 15 min before being transfered to 10% buffered formalin and allowed to incubate at room temperature overnight. Slices were photographed and staining identified and analysed using ImageJ software for every slice to give an overall numerical value for the area at risk and infarct area in relation to the total area for each heart. Control infarct size was 19.4±2.3 % of the total heart area (n=10).

*Immunohistochemistry of the human coronary microvasculature*

Right atrial appendage samples were obtained from patients undergoing coronary artery by-pass grafting at University Hospitals Bristol NHS Foundation Trust. The study complies with the Declaration of Helsinki and was approved by local ethics committee (REC: 10/H0606/36). All participants gave written informed consent. Coronary micro-arteries with smooth muscle layers 1 cells thick and external diameter <200 μm were dissected from the tissue and fixed by cannulating and perfusing with 2% paraformaldehyde at 37 °C, and washing with PBS, and then incubated in blocking buffer (1% BSA and 0.1% Tween 20, pH 7.1) for 60 minutes at 37 °C. Vessels were incubated overnight at 4 °C with primary antibody (Anti-NPY1R, ab183108, 1:200) in the lumen and bath. The micro-artery was washed with PBS and incubated with Alexa Fluor 488 secondary antibody (1:1000 goat antirabbit IgG; A110008) for 2 hours at room temperature. Nuclei were stained with DAPI (1.5 μM) and the elastin with Alexa-Fluor 633 hydrazide (250 nM). Vessels were visualized using an Olympus confocal microscope using a x40 (1.15 NA, 0.25 mm WD) water immersion objective, obtaining z‐stacks through the vessel wall in 1 μm steps with appropriate filters.

*Power calculation and statistical analysis*

Our previous measures of venous NPY in STEMI patients demonstrated an initial value of 17.4 decreasing over 48 hours to 9 pg/ml [^3^](#_ENREF_3). Assuming this magnitude of difference between patients undergoing emergency PCI (for STEMI) compared to elective PCI (for SA and ACS) with a standard deviation of 12 pg/ml, we aimed to recruit at least 43 patients in each group to be 90% powered to detect this difference (alpha of 0.05). Because of the way patients were recruited through the 24-hour cycle of clinical activity, we enrolled 45 patients with STEMI and 48 with SA/ACS. In the process of recruitment, we also identified a subset of patients undergoing elective angiography with NCA (n=16) who are included as a separate group for comparison. Data are presented as mean ± standard deviation, or median [interquartile range] if data did not pass a normality test (D'Agostino Kurtosis). A paired t-test or one-way ANOVA with post-hoc analysis (Bonferroni) was used to determine significance. Non-parametric data from independent groups were compared using a Mann-Whitney-U or Kruskal Wallis one way ANOVA. A Pearson coefficient was used to study correlation of two sets of normal distributed data and a Spearman coefficient for non-parametric data. Mutiple linear regression was used to determine the relationship between three potentially inter-related variables. Discrete data was analysed using Chi-squared or Fisher’s exact test in contingency tables. All significance tests are two-tailed and significance accepted at p<0.05.

**References**

1. Thygesen K, Alpert JS, Jaffe AS, Chaitman BR, Bax JJ, Morrow DA, White HD, Group ESCSD. Fourth universal definition of myocardial infarction (2018). Eur Heart J 2018.

2. Task Force on the management of STsegment elevation acute myocardial infarction of the ESC, Steg PG, James SK, Atar D, Badano LP, Blomstrom-Lundqvist C, Borger MA, Di Mario C, Dickstein K, Ducrocq G, Fernandez-Aviles F, Gershlick AH, Giannuzzi P, Halvorsen S, Huber K, Juni P, Kastrati A, Knuuti J, Lenzen MJ, Mahaffey KW, Valgimigli M, van 't Hof A, Widimsky P, Zahger D. ESC Guidelines for the management of acute myocardial infarction in patients presenting with ST-segment elevation. Eur Heart J 2012;**33**(20):2569-619.

3. Cuculi F, Herring N, De Caterina AR, Banning AP, Prendergast BD, Forfar JC, Choudhury RP, Channon KM, Kharbanda RK. Relationship of plasma Neuropeptide Y with angiographic, electrocardiographic and coronary physiology indices of reperfusion during ST elevation myocardial infarction. Heart 2013.

4. Yamamoto K, Ito H, Iwakura K, Shintani Y, Masuyama T, Hori M, Kawano S, Higashino Y, Fujii K. Pressure-derived collateral flow index as a parameter of microvascular dysfunction in acute myocardial infarction. J Am Coll Cardiol 2001;**38**(5):1383-9.

5. Kohlhauer M, Dawkins S, Costa ASH, Lee R, Young T, Pell VR, Choudhury RP, Banning AP, Kharbanda RK, Oxford Acute Myocardial Infarction S, Saeb-Parsy K, Murphy MP, Frezza C, Krieg T, Channon KM. Metabolomic Profiling in Acute ST-Segment-Elevation Myocardial Infarction Identifies Succinate as an Early Marker of Human Ischemia-Reperfusion Injury. J Am Heart Assoc 2018;**7**(8).

6. Gilard M, Mansourati J, Etienne Y, Larlet JM, Truong B, Boschat J, Blanc JJ. Angiographic anatomy of the coronary sinus and its tributaries. Pacing Clin Electrophysiol 1998;**21**(11 Pt 2):2280-4.

7. Cuculi F, Dall'Armellina E, Manlhiot C, De Caterina AR, Colyer S, Ferreira V, Morovat A, Prendergast BD, Forfar JC, Alp NJ, Choudhury RP, Neubauer S, Channon KM, Banning AP, Kharbanda RK. Early change in invasive measures of microvascular function can predict myocardial recovery following PCI for ST-elevation myocardial infarction. Eur Heart J 2014;**35**(29):1971-80.
